# Supplementary material for: A global synthesis and assessment of free-ranging domestic cat diet
Source: Nat Commun. 2023 Dec 12;14:7809. doi: 10.1038/s41467-023-42766-6 (PMC10716121; doi:10.1038/s41467-023-42766-6)
Supplement: Supplementary file 1 — Supplementary Information [file 41467_2023_42766_MOESM1_ESM.pdf]

A Global Synthesis and Assessment of Free-ranging Domestic Cat Diet

Supplementary Materials

Christopher A. Lepczyk, Jean E. Fantle-Lepczyk, Kylee D. Dunham, Jocelyn Lindner,

Elsa Bonnaud, Tim S. Doherty, John C. Z. Woinarski

Table SI1. Number of species and percent of the total sample within each taxonomic class.

Class	Number of species	% of sample
Aves - Birds	981	47.07
Reptilia - Reptiles	463	22.22
Mammalia - Mammals	431	20.68
Insecta - Insects	119	5.71
Amphibia - Amphibians	57	2.74
Malacostraca - Crustaceans	11	0.53
Arachnida - Spiders	9	0.43
Chilopoda - Centipedes	5	0.24
Actinopterygii - Ray-finned fishes	4	0.19
Gastropoda - Snails and slugs	3	0.14
Diplopoda - Millipedes	1	0.05

Table SI2. Species identified as cat prey items that occurred in ≥ 20 studies.

Species	Common name	Number of studies	Closest continent
Mus musculus	House mouse	158	Africa, Antarctica, Asia, Australia, Europe, N. America, S. America
Oryctolagus cuniculus	European rabbit	114	Africa, Antarctica, Australia, Europe, S. America
Rattus rattus	Black rat	74	Africa, Asia, Australia, Europe, N. America, S. America
Passer domesticus	House sparrow	58	Asia, Australia, Europe, N. America, S. America
Rattus norvegicus	Brown rat	54	Asia, Australia, Europe, N. America, S. America
Sturnus vulgaris	Common starling	42	Africa, Australia, Europe, N. America
Turdus philomelos	Song thrush	33	Australia, Europe
Columba livia	Rock dove	27	Africa, Asia, Australia, Europe, N. America
Myodes glareolus	Bank vole	27	Europe
Chloris chloris	European greenfinch	26	Australia, Europe
Phasianus colchicus	Ring-necked pheasant	26	Australia, Europe, N. America
Microtus agrestis	Short-tailed field vole	26	Europe
Apodemus sylvaticus	Wood mouse	25	Europe
Pseudocheirus peregrinus	Common ringtail possum	21	Australia

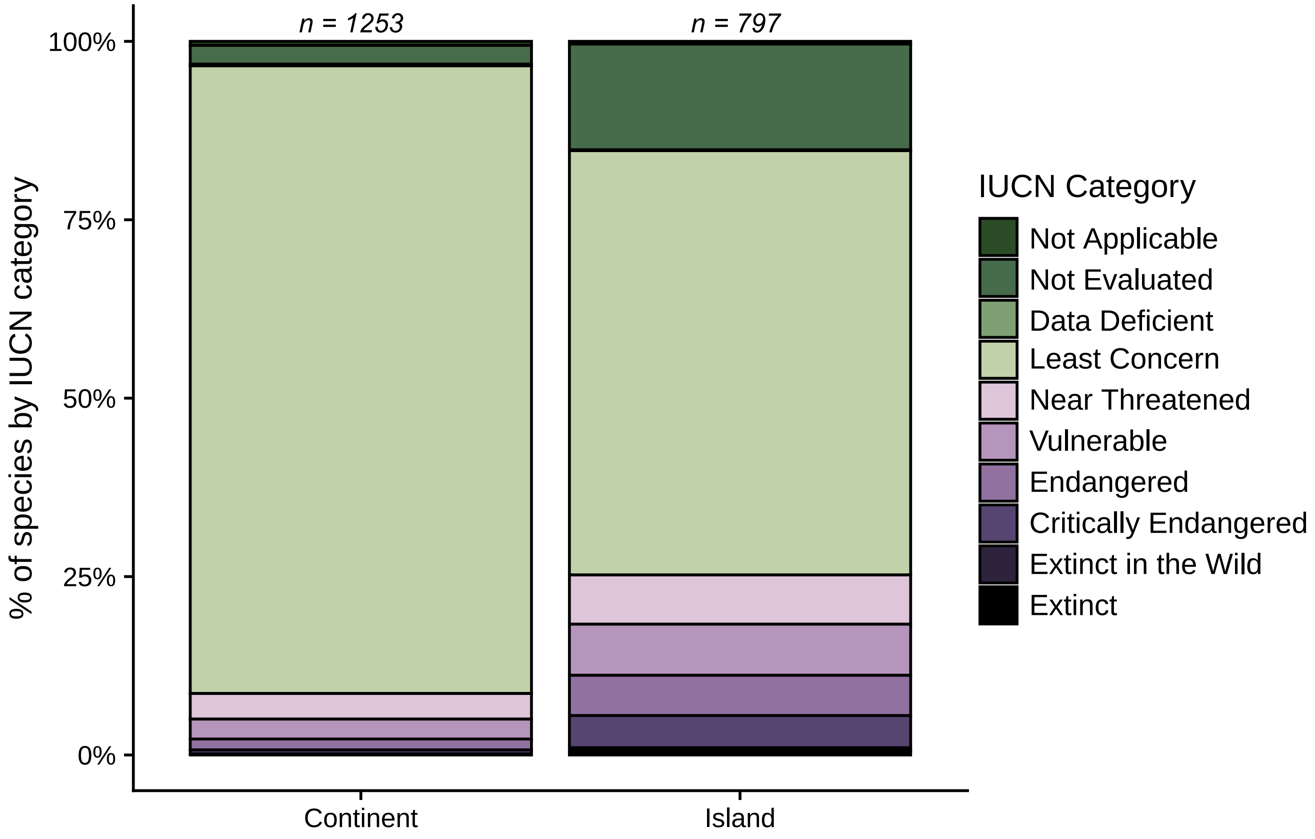

Figure SI1. Percent of species within each IUCN category identified in review of cat diet studies. A total of 1,253 species were identified in studies conducted on continents and 797 species were identified in studies conducted on islands. We note that these numbers do not sum to the total number of unique species identified globally because we only include species identified in studies that explicitly describe being sampled on an island or continent. Further, several species were identified on both islands and on continents.

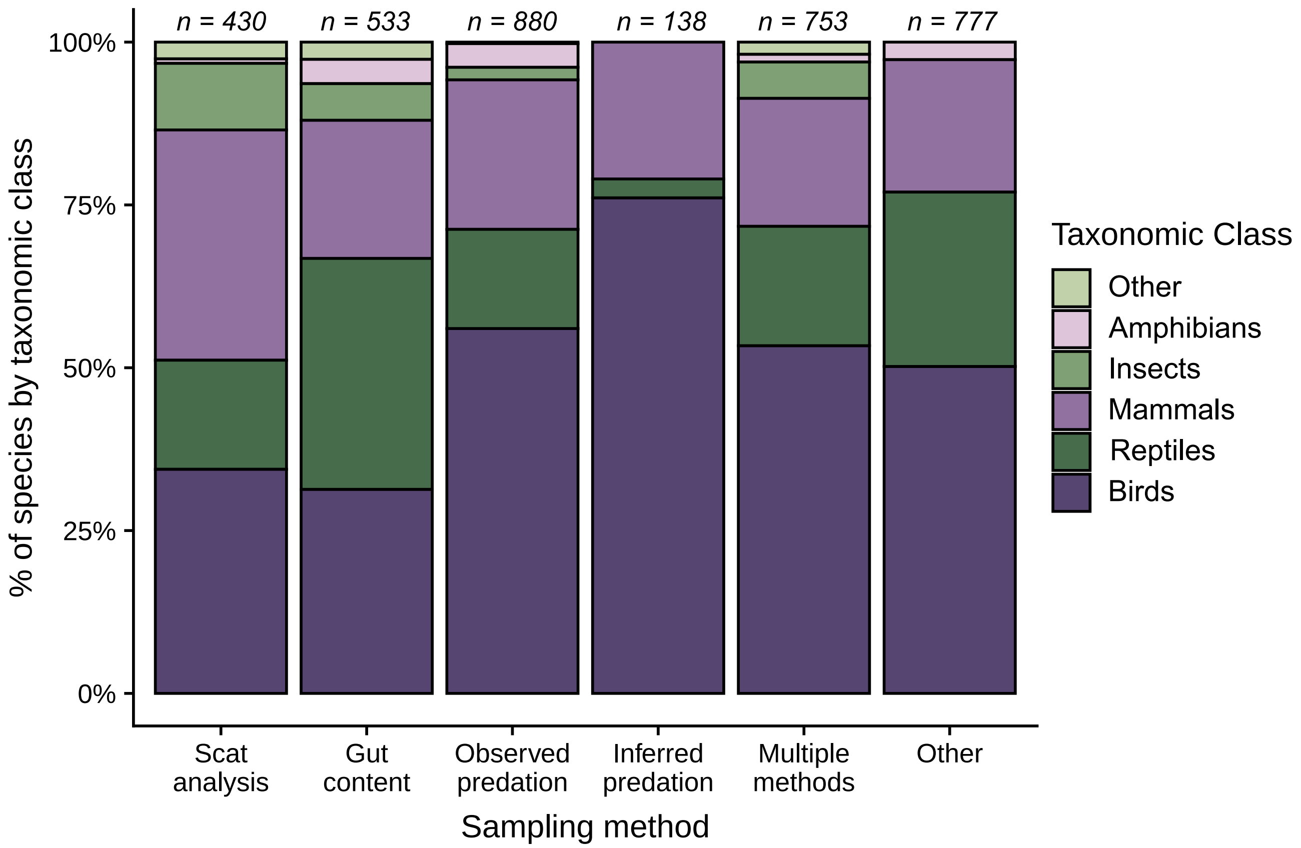

Figure SI2. Taxonomic patterns of species identified as consumed by cats in review of cat diet studies categorized by the sampling method used to identify species. The numbers above each column refer to the number of species identified by each sampling method. We note that these numbers do not sum to the total number of unique species identified globally because many species were identified using several sampling methods.
