# Supplementary material for: A global synthesis and assessment of free-ranging domestic cat diet
Source: Nat Commun. 2023 Dec 12;14:7809. doi: 10.1038/s41467-023-42766-6 (PMC10716121; doi:10.1038/s41467-023-42766-6)
Supplement: Supplementary file 3 — Reporting Summary [file 41467_2023_42766_MOESM3_ESM.pdf]

nature portfolio

Corresponding author(s): Christopher A. Lepczyk

Last updated by author(s): Oct 3, 2023

Reporting Summary

Nature Portfolio wishes to improve the reproducibility of the work that we publish. This form provides structure for consistency and transparency in reporting. For further information on Nature Portfolio policies, see our Editorial Policies and the Editorial Policy Checklist.

Statistics

For all statistical analyses, confirm that the following items are present in the figure legend, table legend, main text, or Methods section.

n/a	Confirmed	
☐	☒	The exact sample size (*n*) for each experimental group/condition, given as a discrete number and unit of measurement
☐	☒	A statement on whether measurements were taken from distinct samples or whether the same sample was measured repeatedly
☒	☐	The statistical test(s) used AND whether they are one- or two-sided<br>*Only common tests should be described solely by name; describe more complex techniques in the Methods section.*
☒	☐	A description of all covariates tested
☒	☐	A description of any assumptions or corrections, such as tests of normality and adjustment for multiple comparisons
☐	☒	A full description of the statistical parameters including central tendency (e.g. means) or other basic estimates (e.g. regression coefficient) AND variation (e.g. standard deviation) or associated estimates of uncertainty (e.g. confidence intervals)
☒	☐	For null hypothesis testing, the test statistic (e.g. *F*, *t*, *r*) with confidence intervals, effect sizes, degrees of freedom and *P* value noted<br>*Give P values as exact values whenever suitable.*
☒	☐	For Bayesian analysis, information on the choice of priors and Markov chain Monte Carlo settings
☒	☐	For hierarchical and complex designs, identification of the appropriate level for tests and full reporting of outcomes
☒	☐	Estimates of effect sizes (e.g. Cohen's *d*, Pearson's *r*), indicating how they were calculated

Our web collection on statistics for biologists contains articles on many of the points above.

Software and code

Policy information about availability of computer code

Data collection	No software was used to conduct the data collection for this study.
Data analysis	All data summaries and analyses (species accumulation curves) were performed in program R version version 4.1.2. Species accumulation curves were generated using "specaccum" function from the "vegan: Community Ecology Package" version 2.5-7in Program R. No custom code was written to perform these analyses.

For manuscripts utilizing custom algorithms or software that are central to the research but not yet described in published literature, software must be made available to editors and reviewers. We strongly encourage code deposition in a community repository (e.g. GitHub). See the Nature Portfolio guidelines for submitting code & software for further information.

Data

Policy information about availability of data

All manuscripts must include a data availability statement. This statement should provide the following information, where applicable:

- Accession codes, unique identifiers, or web links for publicly available datasets
- A description of any restrictions on data availability
- For clinical datasets or third party data, please ensure that the statement adheres to our policy

The cat diet records data used in this study will be made available upon request to the corresponding author via email. The data used to generate figures are

available in the source data Excel file. We included body mass data for birds from https://doi.org/10.6084/m9.figshare.16586228.v7 and for reptiles, amphibians, and mammals from https://doi.org/10.6084/m9.figshare.10075421.v1. Species icons used in figures 1 and 4 were accessed from http://phylopic.org.

Human research participants

Policy information about studies involving human research participants and Sex and Gender in Research.

Reporting on sex and gender	Not applicable
Population characteristics	Not applicable
Recruitment	Not applicable
Ethics oversight	Not applicable

Note that full information on the approval of the study protocol must also be provided in the manuscript.

Field-specific reporting

Please select the one below that is the best fit for your research. If you are not sure, read the appropriate sections before making your selection.

☐ Life sciences ☐ Behavioural & social sciences ☒ Ecological, evolutionary & environmental sciences

For a reference copy of the document with all sections, see nature.com/documents/nr-reporting-summary-flat.pdf

Ecological, evolutionary & environmental sciences study design

All studies must disclose on these points even when the disclosure is negative.

Study description	Our study examined patterns of free-ranging cat diet.
Research sample	We compiled information on cat diet through searches of both peer-reviewed and gray literature in Google Scholar and Web of Science using the keywords 'cat predation,' 'feral cat,' 'cat diet,' and 'Felis catus.' From each source we evaluated if it contained data on cat diet or predation as well as reviewed its reference section for additional unique articles or databases pertaining to cat diet and predation. We iteratively evaluated any new sources identified in the references for both data and additional articles or databases in an exhaustive manner (i.e., each manuscript's references were found and evaluated for diet if they were related to cats). After this exhaustive search we identified 544 unique publications (books, journal articles, theses, and agency reports) that contained data on cat diet. Records that were not identified to the species level (i.e., only to genus) were removed from the analysis, leaving us with 533 unique publications with records of species consumed by cats. For the purposes of this research, when records were identified to the subspecies level, we reclassified them to the species level.
Sampling strategy	Sample sizes were determined by counting the number of unique records within specific categories. For example, we tallied the number of unique species identified across all studies that we found during our literature review.
Data collection	Publications include specific search terms were collected over a number of years. Researchers read through each publication and recorded relevant information into an excel spreadsheet.
Timing and spatial scale	The database contains all literature that was available through May 2021.
Data exclusions	Records were excluded from the data when species were not recorded to at least the species level (i.e., species was unknown but researchers may have recorded Family or Genus).
Reproducibility	Analyses were run several times (e.g., species accumulation curves) and each attempt produced the same results.
Randomization	This is not relevant to our study because the groups we considered (e.g., taxonomic class) are known constants.
Blinding	Blinding data were not relevant to our study because we did not perform any type of experiment.

Did the study involve field work? ☐ Yes ☒ No

Reporting for specific materials, systems and methods

We require information from authors about some types of materials, experimental systems and methods used in many studies. Here, indicate whether each material, system or method listed is relevant to your study. If you are not sure if a list item applies to your research, read the appropriate section before selecting a response.

Materials & experimental systems

n/a	Involved in the study
☒	☐ Antibodies
☒	☐ Eukaryotic cell lines
☒	☐ Palaeontology and archaeology
☒	☐ Animals and other organisms
☒	☐ Clinical data
☒	☐ Dual use research of concern

Methods

n/a	Involved in the study
☒	☐ ChIP-seq
☒	☐ Flow cytometry
☒	☐ MRI-based neuroimaging
